# Body Composition Changes in Male and Female Elite Soccer Players: Effects of a Nutritional Program Led by a Sport Nutritionist

**DOI:** 10.3390/nu16030334

**Published:** 2024-01-23

**Authors:** Cristian Petri, Luca Pengue, Alice Bartolini, Duccio Pistolesi, Luis Suarez Arrones

**Affiliations:** 1 Department of Sport and Informatics, Section of Physical Education and Sport, Pablo de Olavide University, 41013 Sevilla, Spain; cpet2@alu.upo.es; 2 A.C.F. Fiorentina S.r.l., 50137 Florence, Italy; lpengue@acffiorentina.it (L.P.); alib06@hotmail.it (A.B.); dpistolesi@acffiorentina.it (D.P.)

**Keywords:** soccer, skinfold thickness, nutrition, eating habits

## Abstract

Background: Soccer is a game in constant evolution and the intensity of play is increasing. Nutrition can play a role in the physical performance of elite players, maintaining their health and facilitating recovery. It is important to cover players’ energy demands, and low energy availability may therefore result in impaired performance. This study aimed to evaluate alterations in body composition to determine the effects of a nutritional program led by a sport nutritionist. Methods: A group of 88 elite soccer players from a Serie A club in Italy (44 males aged 26.5 ± 3.0 years and 44 females aged 27.1 ± 5.2 years) were enrolled. To evaluate changes in body composition, bioimpedance and anthropometric measurements were obtained following the protocol of the International Society for the Advancement of Kinanthropometry (ISAK). Results: Compared with females, males had more muscle mass and less fat mass in both seasons evaluated. Comparing the first and last seasons, the male soccer players showed increased muscle mass and decreased fat mass while the female soccer players only showed decreased fat mass. Conclusions: The presence of a specialist sport nutritionist on the staff of professional soccer clubs could be important to ensure energy availability and evaluate body composition during the season.

## 1. Introduction

Football (soccer) is a game in constant evolution in both physical and technical parameters, with a substantial increase during match-play in the number of occasions involving high-intensity running [1]. Training regimens have become more physically demanding in an attempt to prepare players to cope with these high-intensity demands and address individual player needs. In recent decades, the application of sport science to improve athletes’ health and performance has become routine in most team sports [2]. However, recent research has highlighted the relevance of invisible strategies (i.e., those other than training plans) in optimizing athlete health and performance [3]. Nutrition is considered a key invisible strategy, which can play a valuable role in optimizing the physical and mental performance of elite players during training and match-play, maintaining their overall health throughout a long season and facilitating recovery from injury [4].

Most team sport studies focusing on athlete nutrition have reported on the health- and performance-related positive effects of diets [5,6] and supplementation [7,8,9]. However, some promising data have emerged regarding the efficacy of nutrition education interventions in team sports [10]. Nutrition education interventions are specific programs designed to assist target populations in modifying their eating habits and/or enhancing their nutrition knowledge [11]. Improved eating habits seem to be a key strategy in optimizing football players’ performance and health [12,13]. However, while the exponential rise in sports nutrition research during recent years has advanced our knowledge and expertise, it brings with it confusion as to what is sound advice. Those providing sports science support at the elite level should follow an evidence-based approach [14], but it is often difficult to interpret the available evidence and make sense of the controversies that may exist, given the influx of noise from social media channels [15].

Another topic of interest is the prevalence of low energy availability (LEA) in female and male athletes is high, often caused by congested match schedules [16,17]. Kick-off times have become more variable, with teams required to play early or late to accommodate television schedules. The travel required to compete in multiple domestic and international tournaments add to the demands on players, with different logistical challenges depending on the geographical location. Difficulties in eating well while travelling, poor appetite, or the stress caused by matches can affect players after competition by hindering their post-match recovery and affecting their energy levels. Low levels of energy negatively affect muscle recovery, muscle mass, and neuromuscular function, increasing the risk of injuries and illness that may negatively affect performance [18]. Achieving or maintaining non-optimal lower lean body mass and fat levels through long-term LEA may therefore result in impaired health and performance, as proposed in the Relative Energy Deficiency in Sport model [19]. Treatment for athletes is performed primarily to increase energy availability and evaluate body composition during the season, and requires a team approach including a nutritional program run by a sport nutritionist.

Body composition assessment is commonly used to track the improvement of elite football players throughout the regular season. Body fat mass is generally estimated in elite soccer players by assessment methods such as surface anthropometry (skinfolds), bioelectrical impedance analysis, and dual-energy X-ray absorptiometry (DXA). Among the methods designed for assessing body composition, surface anthropometry and the bioelectrical impedance analysis (BIA) represent two useful tools, particularly in field contexts where elaborate laboratory techniques may not always be feasible [20], while DXA is associated with logistical and high financial costs that might prevent its frequent use in the majority of professional the soccer teams. Anthropometry involves the measurement of various body dimensions and proportions, providing insights into an individual’s physique. Common anthropometric measurements include stature, body mass, skinfold thickness, and circumferences of specific body parts. By utilizing standardized protocols and instruments, practitioners can calculate body fat percentage, lean body mass, and other relevant parameters for soccer players. Particularly, anthropometry is renowned for its simplicity, cost-effectiveness, and non-invasiveness, making it ideal for large-scale studies, sports assessments, and community health evaluations. These measurements can be performed with minimal equipment and just training, allowing for rapid assessments even in resource-limited environments [21]. When implementing dietary changes, monitoring raw anthropometric data provides a direct and immediate assessment of alterations in body dimensions. Regular measurements, such as skinfold thickness, circumferences, and somatotype allow for sports nutritionists and coaches to track changes over time. This real-time feedback is particularly relevant in the dynamic and physically demanding environment of soccer, where players’ nutritional needs can vary based on training intensity, match schedules, and individual metabolic responses. Additionally, anthropometry in soccer often involves the use of predictive equations derived from anthropometric measurements to estimate specific body composition components. These equations take into account variables such as skinfold thickness, body circumferences, and body density to calculate parameters like body fat percentage and lean body mass. While raw data provides immediate feedback, predictive equations offer a more in-depth analysis, allowing for practitioners to understand the nuanced changes in body composition resulting from dietary modifications [22,23]. Similarly, the bioelectrical impedance analysis (BIA) is a method used to assess body composition by measuring the impedance or opposition to the flow of an electric current as it passes through body tissues. BIA is based on the principle that lean tissue, which contains a higher water and electrolyte content, conducts electrical current more easily than fat tissue. BIA involves passing a low-level electrical current through the body and measuring the impedance to the flow of this current. As for the anthropometry, with BIA, it is possible to consider raw parameters, such as resistance, reactance, and phase angle, or to predict body mass components using predictive equations. Both approaches—monitoring raw data and using predictive equations—have been successfully employed in the literature within the soccer context [24,25]. Regarding the raw data, the bioelectrical phase, in particular, can provide info regarding aerobic power [26]. The dynamic nature of the sport requires a flexible and multifaceted approach to assess the impact of diet on players’ bodies. Studies often utilize a combination of methods to obtain a comprehensive understanding of how dietary interventions influence body composition, performance, and overall health [27].

This study aimed to evaluate the differences in body composition using surface anthropometry and BIA in male and female soccer players to determining the effects of a nutritional program led by a sport nutritionist in an elite soccer club (following the UEFA rules such as “Food first” and personalized supplementations [13]) with mandatory breakfasts and meals at the club, in conjunction with individual interventions.

## 2. Materials and Methods

### 2.1. Participants

This study involved a group of 88 international-level, elite male (n = 44) and female (N = 44) soccer players belonging to four senior squads of a Serie A club in Italy (Table 1). Eighteen female soccer players playing in Serie A during the 2015 season (FS15) and 26 female soccer players playing in Serie A during the 2023 season (FS23) were compared. Seventeen male soccer players playing in Serie A during the 2015 season (MS15) and 27 male soccer players playing in Serie A during the 2023 (MS23) season were also compared. All the players evaluated had played several times during their career with their respective senior national teams (8 nationalities). Data were collected in the club at the end of the domestic competition season (i.e., Serie A).

All the players trained during the season, completing approximately 8–9 h of on-field soccer training plus 1 or 2 competitive matches per week. Data came from routine monitoring over the season; therefore, institutional ethics committee authorization was not required [28]. The study conformed with the current national and international laws and regulations governing the use of human participants. The Antidoping Lab Institutional Review Board (Qatar) conformed to the recommendations of the Declaration of Helsinki approved the present study (IRB number: E201300004).

### 2.2. Body Composition

#### 2.2.1. Skinfold Thickness

Participants presented in a rested, fasted, and hydrated state and were instructed to follow standard protocols of food and fluid, to finish their last meal at least 2.5 h before the measurement, and to arrive with an empty bladder. They were instructed to avoid strenuous exercise, alcohol, stimulants, or depressants for 24 h prior to testing, as well as to remove all metal objects and wear minimal clothing (underwear only) during the assessments. All participants verbally agreed to the testing conditions. Each session was conducted at the same hour of the day, and in the same well-ventilated room with controlled temperature and humidity. The methodology used for the assessment of body composition was in accordance with our previous studies [21,25], using the integration of anthropometry, skinfold thickness, and bioimpedance.

Body mass was taken to the nearest 0.1 kg with an electronic scale (OHAUS Corp., Florham Park, NJ, USA); stature was measured with a stadiometer (Seca 213, Hamburg, Germany) to the nearest 0.5 cm. BMI was calculated as body mass (kg)·height^2^ (m). Seven skinfolds thicknesses (triceps, subscapular, biceps, suprailiac, abdominal, thigh, and medial calf) were measured and the sum of all measurements was calculated as reported in the literature [22]. All skinfolds and height measures were taken by the same Level III International Society for the Advancement of Kinanthropometry qualified anthropometrist according to standard methods [21], whose technical error was 5% and 1.5% for skinfolds and all other measurements, respectively. Duplicate measurements were obtained from each site and the mean of the two first measures was used for analysis. A third measurement was taken if the technical error of measurement was exceeded and, consequently, the median of the triplicate measurements was used for subsequent analysis. FM% was then obtained using the soccer-specific formula proposed by Suarez-Arrones et al. [22] in males and the formula proposed by Durnin and Womersley [29] in females.

#### 2.2.2. Whole-Body Bioimpedance Analysis (BIA)

Whole-body impedance (BIA 101 Sport Edition, BIVA^®^ PRO, Akern^®^, Florence, Italy) was generated in soft tissues to oppose the flow of an injected alternate current and was measured from skin Ag/AgCI electrodes placed at a fixed distance (5 cm) on the hands and feet (whole-body analysis). The device emitted an alternating sinusoidal electric current of 400 mA at an operating single frequency of 50 kHz (±0.1%). Resistance (R, O) is the opposition to the flow of an injected alternating current, at any current frequency, through intra- and extracellular ionic solutions, while reactance (Xc, O) is the dielectric or capacitive component of cell membranes, organelles, and tissue interfaces. Starting from these parameters, an estimate of the following body compartments parameters, such as body cellular mass (BCM in kg) and fat mass (%), was derived using the formulas proposed by Kotler et al. [30] and Campa et al. [31], respectively, in males. Female soccer players were assessed using the Kotler et al. [30] and Matias et al. formulas [32] for BCM and FM, respectively. An additional parameter was body cell mass index (BCMI) in kg/h^2^ in order to index for the different gender heights.

### 2.3. Statistical Analysis

Data are presented as mean ± standard deviation (SD). The Shapiro–Wilk test was used to verify that all the variables showed a normal distribution. A one-way analysis of variance (ANOVA) was used to determine differences between squads. In the event of a significant difference, Bonferroni’s post hoc tests were employed to identify any localized effects. The standardized difference or effect size (ES, 95% confidence interval [95% CI]) in the different variables was calculated. Threshold values for assessing magnitudes of the ES were <0.20, 0.20, 0.60, 1.2, and 2.0 for trivial, small, moderate, large, and very large, respectively [33].

## 3. Results

**Figure 1:**
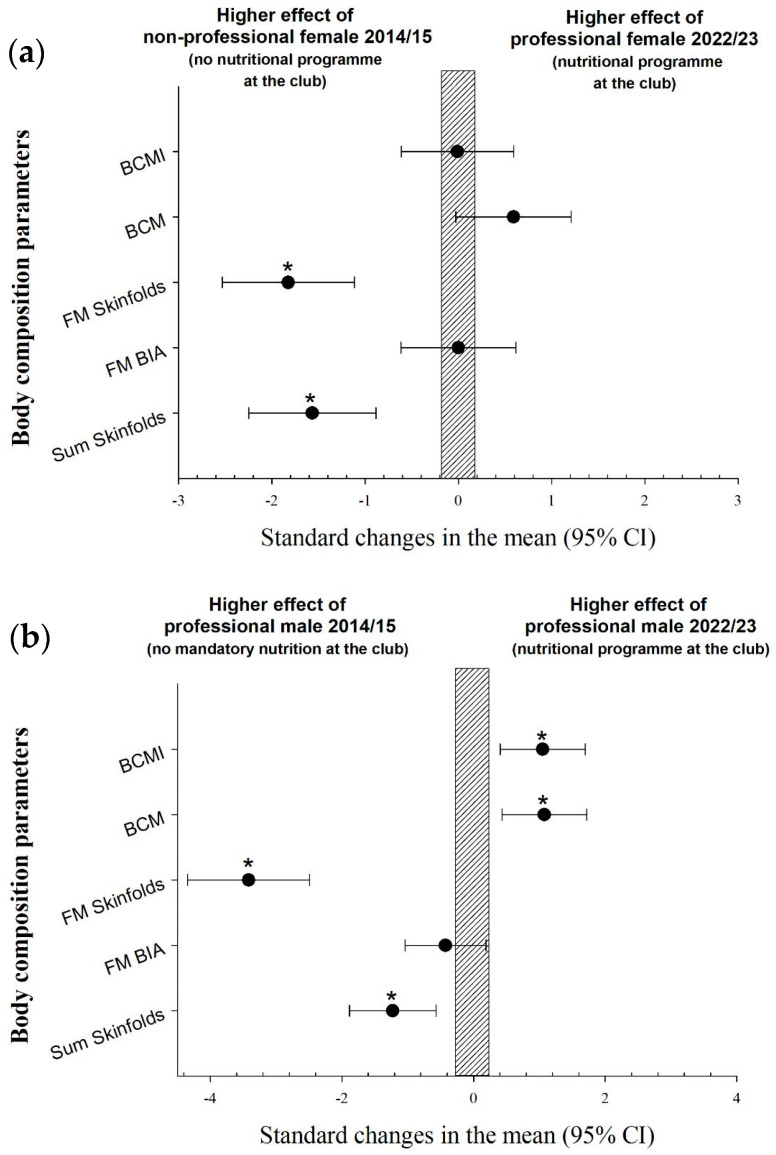
(**a**) The differences in body composition between female teams. (**b**) The differences in body composition between male teams. * *p* < 0.05. BCM: body cellular mass; BCMI: body cellular mass index; FM: percentage of fat-mass.

Descriptive values of the different parameters derived from the anthropometric and BIA assessments are shown in Table 2. The standardized differences between squads are illustrated in Figure 1a,b.

FS15 showed a significantly higher ∑7 SKF and FM% estimated from skinfolds in comparison with FS23 (*p* < 0.05), while there were no differences between female teams in BCM, BCMI, and FM% estimated from BIA (Figure 1a). MS15 showed a significantly higher ∑7 SKF and FM% estimated from skinfolds than MS23 (*p* < 0.05), while there were no differences between male teams in FM% estimated from BIA. BCM and BCMI were significantly higher in MS23 in comparison with MS15 (*p* < 0.05, Figure 1b).

## 4. Discussion

The aim of this study was to evaluate the differences in body composition in male and female soccer players to determine the effects of a nutritional program led by a sport nutritionist in an elite soccer club with mandatory breakfasts and meals at the club, in conjunction with individual interventions. Our hypotheses on the need for a nutrition program were created when we found, during the 2014–2015 season, that the dietary habits of a male first-division team did not follow the international guidelines in terms of macronutrients and energy and that, consequently, there was a decrease in the muscular component during the season [34], especially after several congested periods of play. The main findings of the present study showed the substantial positive effects of the nutritional program in a professional soccer club in both female and male players.

In female soccer, as in male soccer and other sports, club medical staff use body composition assessment to aid interpretation of health- and performance-relevant results and inform subsequent exercise and dietary interventions [35]. Ensuring that assessment is useful and safe for players requires practitioners to be aware of possible problems that can occur when the focus on body mass and body composition is over-emphasized [34]. Anthropometric measurements that are taken should follow a protocol such as that of the International Society for the Advancement of Kinanthropometry (ISAK), and all practitioners who work in a sport should be fully qualified to ensure the accurate interpretation of the data collected. These measurements have been identified as appropriate methods for detecting body composition changes due to an intervention [36].

Comparing our data collected during the 2014/2015 season from nonprofessional female soccer players with other similar studies in the scientific literature, the results were similar in terms of body fat % (16.0% of fat mass) [37,38]. If we compare the data collected with that of Santos et al. regarding the sum of skinfolds, the data for the 2014/2015 season are around the mean of the 25th percentile, while the data for the 2022/2023 season lie in the lower part of the 25th percentile. This is due to the development of female soccer over the last decade in terms of financial support, professionalism, and consequent growth in the level of participation. Despite recent progress, there is considerably less research on female soccer players than male, including in the field of body composition assessment; therefore, it is very difficult to make comparisons. In contrast, there are multiple body composition references for male soccer players [39,40].

Body composition is crucial for achieving an optimal physical level, which can translate into a good level of play because performance in soccer depends on multiple technical, biomechanical, tactical, mental, and physiological factors [13,41]. During soccer practice, there are a multitude of movements that are affected by weight and muscle power, such as accelerations, decelerations, changes in direction, and vertical jumps, so muscle strength, impact loading, and an optimal level of body fat are key determinants in physical preparation to improve performance in soccer [42,43].

The physical demands on elite soccer players have been increasing in recent decades, not only in the amount of training and/or competition, but also in the intensity of effort during matches and shorter recovery periods between competitions or training sessions [44,45]. It is logical to think that body composition has evolved over time, following the evolution of the game. This could be the reason for the differences found in our sample in terms of the sum of skinfolds and BCM. Recently, Moya-Amaya et al. verified the somatotype trend in the last few decades in professional male soccer players, observing a decrease in the endomorphic component, evolving from balanced mesomorphy to ecto-mesomorphy [46].

Sometimes the mismatch between energy intake and exercise energy expenditure that causes LEA in athletes may be caused intentionally to decrease body mass or optimize body composition for competition, or to avoid weight gain during an injury process. Several potential reasons may explain inadvertent LEA, such as large energy needs and suppressed appetite during periods of high-intensity training, especially when combined with adherence to ultra-healthy or “clean” eating with low energy density diets [47,48]. It has been suggested that long-term LEA could negatively affect sport performance through indirect mechanisms, such as reduced recovery and impairment of optimal muscle mass and function [49]. Since LEA increases the risk of injury and illness, performance may also be impaired by loss of training time [49].

Based on our previous investigations [50], achieving optimal performance in sport requires medical supervision, including that of a sports nutritionist, to ensure periodizing of energy, fuel availability, and an adequate protein intake around key training sessions and matches. Specifically, to reduce the risk of LEA and to ensure optimal nutritional habits, professional soccer clubs include nutritional programs, including on their staff a sport nutritionist prescribing mandatory meals for training days (breakfast, post-training, and lunch) and individual protocols for supplementations for each athlete. The results of the present investigation showed substantial improvements in body composition with the introduction of a nutritional program and a nutritionist for the men’s and women’s teams. The sum of the skinfolds and FM% were higher in both teams (female (31%) and male (19%)) before the implementation of the nutrition program with mandatory meals at the club, and lean mass was higher at the end of the season when the nutrition project was mandatory for male players due to better nutrition in addition to the possible influence of difference in strength training (Table 2, Figure 1).

In the women’s team during the 2014–2015 season, in addition to not having a nutritional program at the club, women’s football in Italy was not professional. This probably also had an influence on having higher levels of body fat, with the sum of both skinfolds and the FM% (from skinfolds) being significantly higher than in the 2022–2023 season, while there was no difference in the FM% estimated using bioimpedance (Figure 1). These results confirm that the use of bioimpedance for the evaluation of fat mass is inadequate because it underestimates the results and its sensitivity to change, as has been previously reported in the scientific literature [22]. However, in the female team, lean mass did not increase with the nutrition program in terms of BCM and BCMI. This could be dictated by the fact that women’s football is in evolution in Italy, and we also have to consider strength training, which was not strictly controlled during both seasons. The decreases in the sum of skinfolds and in the FM% bring these data closer to those reported in the literature [21].

**Figure 2:**
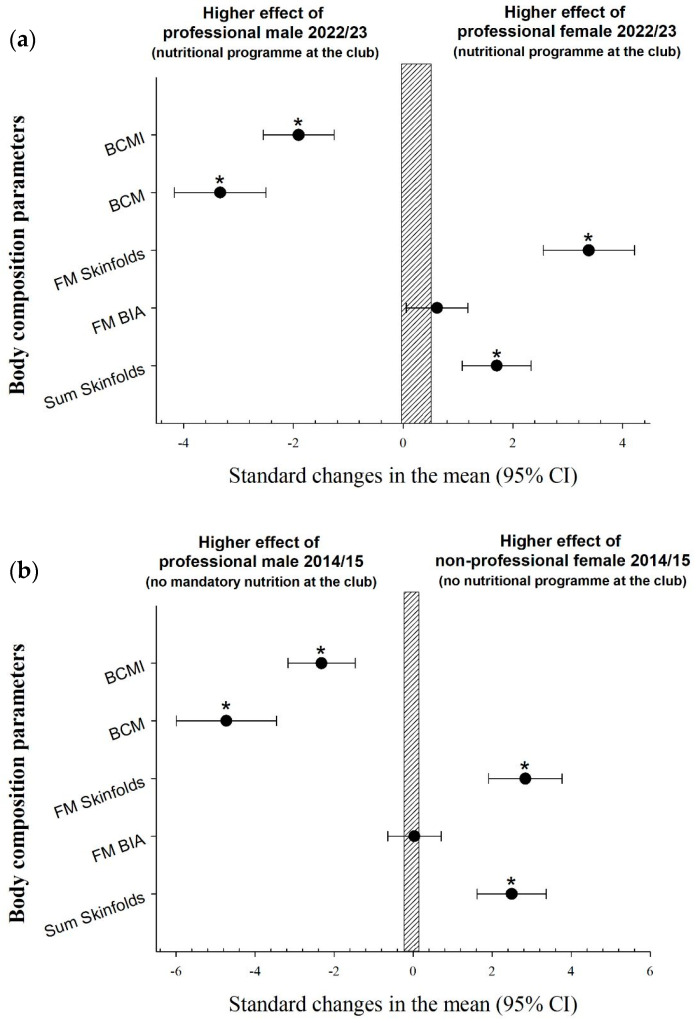
(**a**) The differences in body composition between professional male vs. female teams. (**b**) The differences in body composition between professional male (without intervention) vs. nonprofessional female teams. * *p* < 0.05. BCM: body cellular mass; BCMI: body cellular mass index; FM: percentage of fat-mass.

In relation to the developments in the male team, our data showed a clear impact on the reduction in body fat (skinfolds) and increase in muscle mass when mandatory breakfast and lunch were introduced at the club and the body composition of the players was monitored regularly. As with the women, there were no differences in the FM% estimated with bioimpedance (Figure 1), confirming again that the use of bioimpedance for the evaluation of fat mass and its sensitivity to change is questionable [22]. The same goes for the comparison between men and women. Male soccer players showed statistically lower values for the sum of skinfolds and FM% estimated from skinfolds with higher values of lean mass [21], while there were no differences in FM% estimated with bioimpedance (Figure 2).

The present study has limitations. An important limitation is not knowing the exact strength training program that the players performed throughout the whole season, as well as the training load performed on the field. This could have substantially influenced the results, especially for lean mass. As in other studies, we do not have this information in detail, as it was collected by two different sets of staff in two different seasons, although the working methodology in both cases was very similar. In addition, the data obtained do not include nutritional health studies’ results, so it is proposed to include these in future studies. Currently, there are only specific equations available for estimating body composition in male football players. Future research involving female elite football players should be designed to develop specific equations for estimating fat free mass (FFM) and fat mass (FM).

## 5. Conclusions

A nutrition program led by a specialized sport nutritionist with mandatory breakfasts and meals at the club, in conjunction with individual interventions, should be included by professional soccer clubs to improve body composition in professional female and male soccer players. A sport nutritionist will manage, prevent, and/or detect different deficiencies caused by wrong choices, quantities of food, and different nutrition cultures, encouraging the acquisition of good habits, avoiding food fads, and implementing personalized supplementation. A balanced dietary habit in athletes will help to improve performance, aid in injury and illness prevention, and provide habits and benefits that last well beyond the end of their careers. Therefore, these are benefits both for individual players and for the football club. Finally, the findings of this observational study can serve as valuable reference data for professionals in the soccer community who are interested in monitoring changes in body composition using field methods such as anthropometry and BIA. Anthropometry appears to be the best alternative to dual-energy X-ray absorptiometry when a limited time and budget are available. The application of soccer-specific formulas to estimate the percentage of fat mass is highly recommended for these methods, enhancing their relevance and applicability in the context of sport. The use of sum of skinfolds appears to be a very good alternative as a direct and simple parameter for determining changes in FM throughout the season.

## Figures and Tables

**Table 1:** Characteristics of the four groups of participants.

Variable	Professional Female 2022/2023 (n = 44)	Professional Male 2022/2023 (n = 44)	No Professional Female 2014/2015 (n = 44)	Professional Male 2014/2015 (n = 44)
Age (years)	27.0 ± 5.0	26.2 ± 2.9	27.3 ± 5.2	26.8 ± 3.1
Body mass (kg)	62.2 ± 5.7	80.9 ± 7.7	61.0 ± 7.7	77.9 ± 6.6
Height (m)	1.69 ± 0.05	1.84 ± 0.05	1.69 ± 0.07	1.82 ± 0.07

**Table 2:** Descriptive values of the variables.

Variable	Professional Female 2022/2023 (n = 44)	Professional Male 2022/2023 (n = 44)	No Professional Female 2014/2015 (n = 44)	Professional Male 2014/2015 (n = 44)
Sum 7 SKF (mm)	61.4 ± 11.8 ^b,c^	44.0 ± 4.9 ^c,d^	88.6 ± 17.4 ^d^	54.5 ± 6.9 ^b,c^
Fat-Mass skinfolds (%)	12.1 ± 2.1 ^b,c,d^	6.9 ± 0.5 ^a,c,d^	16.6 ± 2.8 ^a,b,d^	10.1 ± 1.4 ^a,b,c^
Rz (Ohm)	536.7 ± 56.2 ^b,d^	436.0 ± 43.2 ^a,c^	554.7 ± 50.3 ^b,d^	468.6 ± 31.4 ^a,c^
Xc (Ohm)	69.8 ± 8.7	65.8 ± 11.1	69.0 ± 6.1	65.0 ± 4.7
BCM (Kg)	29.9 ± 3.2 ^b,d^	45.4 ± 5.7 ^a,c,d^	28.2 ± 2.6 ^b,d^	40.4 ± 2.7 ^a,b,c^
BCMI (Kg/m^2^)	10.4 ± 0.8 ^b,d^	13.4 ± 1.3 ^a,c,d^	10.0 ± 1.0 ^b,d^	12.3 ± 0.9 ^a,b,c^
Phase angle (degree)	7.4 ± 0.6 ^b^	8.6 ± 1.5 ^a,c^	7.1 ± 0.5 ^b^	7.9 ± 0.5
Fat-Mass BIA (%)	12.8 ± 3.9	10.4 ± 3.7	12.8 ± 3.9	12.0 ± 3.0

∑7 SKF = triceps, subscapular, biceps, suprailiac, abdominal, thigh, medial calf. ^a^ Significant differences with professional female soccer players during the season 2022/2023; ^b^ significant differences with professional male soccer players during the season 2022/2023; ^c^ significant differences with nonprofessional female soccer players during the season 2014/2015; ^d^ significant differences with professional male soccer players during the season 2014/2015.

## Data Availability

Data can be obtained from Cristian Petri on a reasonable request cpet2@alu.upo.es.
